# Evaluating Growth in Dry Socket Publications: A Bibliometric Analysis

**DOI:** 10.7759/cureus.78161

**Published:** 2025-01-28

**Authors:** Manju Philip, Ikram UI Haq, Bandar AlMutairi, Saad Bin Shabib, Muhannad A Alshehri, Ibrahim Almuhanna

**Affiliations:** 1 Department of Maxillofacial Surgery and Diagnostic Sciences, College of Dentistry, King Saud Bin Abdulaziz University for Health Sciences, Riyadh, SAU; 2 Department of Information Management, College of Dentistry, King Saud Bin Abdulaziz University for Health Sciences, Riyadh, SAU; 3 Ministry of National Guard Health Affairs, King Abdullah International Medical Research Center, Riyadh, SAU; 4 Internship Unit, College of Dentistry, King Saud Bin Abdulaziz University for Health Sciences, Riyadh, SAU

**Keywords:** alveolar osteitis, bibliometric, dry socket, publication, research productivity

## Abstract

Dry socket, a common postextraction complication, occurs when the blood clot in the tooth socket fails to form or is dislodged, leading to severe pain and delayed healing. This study aimed to analyze the publication trends and key features of dry socket research published from 1905 to 2024 worldwide. A quantitative bibliometric approach was employed to extract data from the Web of Science database. The search strategy included Topics = (“dry socket” OR “alveolar osteitis”) and covered publications up to December 31, 2024. The search captured all types of documents to provide a comprehensive overview. The study analyzed and extracted several bibliometric parameters, including the classification of clinical versus nonclinical studies, level of evidence, trends in research, and citations over time, as well as information on the leading journals, countries, institutions, authors, and top keywords. Data analysis was conducted using Microsoft Excel (version 16, Microsoft Corporation, Redmond, WA) and VOSviewer (version 1.6.10, Centre for Science and Technology Studies, Leiden University, The Netherlands) software. Our search identified 713 documents, averaging 18.85 citations per publication. Most studies were clinical, and level 2 was the most frequently observed level of evidence. Approximately 82% of the publications were released in the past 24 years, from 2001 to 2024. Over one-third of the literature was published in the top 10 journals, with the Journal of Oral and Maxillofacial Surgery being the most preferred. The United States produced the most research and had the highest citation impact. The Saveetha Institute of Medical and Technical Sciences contributed the largest number of publications, while The University of Manchester had the highest citation impact. Majid Eshghpour was the most productive author. The most influential keywords identified were “dry socket”, “alveolar osteitis”, and “tooth extraction”. This bibliometric study provides valuable insights into the evolving body of research on dry sockets, offering a clear picture of the leading topics, influential journals, and key contributors shaping this field. Future research efforts should continue to build on these findings, focusing on innovative clinical interventions, preventive measures, and further exploration of less studied areas.

## Introduction and background

Dry socket, or alveolar osteitis, is a painful complication that can follow tooth extractions, particularly with lower wisdom teeth. It occurs when the blood clot that protects the healing socket gets dislodged or breaks down too early, exposing the bone and nerves. The risk of developing a dry socket is higher with impacted wisdom teeth (30%-38%) compared to routine extractions (0.5-5%), and factors like smoking, poor hygiene, trauma during extraction, and hormonal changes can increase the likelihood [1,2]. Prevention focuses on antiseptic mouth rinses, careful surgical techniques, and good aftercare, though no method guarantees it. Treatment typically targets pain relief, with emerging regenerative options like platelet-rich plasma showing potential. Further research into genetic and biological factors could lead to better prevention strategies and personalized treatments for improved patient outcomes [2-4].

The term dry socket or alveolar osteitis is primarily related to the oral and maxillofacial surgery subspecialty of dentistry [5,6]. Almas et al. examined the 619 papers on tooth extraction socket preservation published from 1968 to 2020. Most papers were published during the last 10 years of study, and the United States emerged as the most productive country (n = 201; 32.47%). The analysis found that terms like alveolar ridge augmentation, extraction sockets, and grafts were often used in 20th-century studies [7].

Falci et al. analyzed the bibliometric characteristics of 100 top-cited papers on the field of third molar surgery. The study highlighted the most common keywords. The University of Barcelona was the most prolific institution in the field of third molar research, while the United States was the top contributor [8]. Cai et al. analyzed 950 articles on oral and maxillofacial neuralgia published from 2004 to 2023. The study showed a rising trend in publications, with most coming from developed countries, and highlighted trigeminal neuralgia, gamma knife radiosurgery, and botulinum toxin as most occurred research themes [9]. Zhao et al. examined the publication trends and advancements in platelet-rich fibrin research, analyzing 946 papers. The majority of the research was conducted in China and the United States. Key recurring keywords included “growth factor”, “platelet-rich plasma”, and “bone regeneration”. The study highlighted that manufacturing processes and new applications are emerging as key research hotspots. These findings provide valuable insights for scholars looking to drive further innovation and exploration in the field of platelet-rich fibrin [10].

A recently published article by Saraç et al. evaluated the quality and bibliometric attributes of research on maxillary sinus augmentation, a procedure designed to enhance vertical bone volume in the maxillary posterior region. The analysis was based on articles retrieved from the Web of Science (WoS) database. The study revealed that Wang HL was the most frequently cited author, and the most common keyword was “dental implants”. The study highlighted the critical role of clinical and patient-focused research in advancing sinus lift techniques [11].

In the 21st century, scholarly publications have experienced significant growth across various fields, including Oral and Maxillofacial Surgery [12,13]. Bibliometric analysis offers a valuable method for quantitatively assessing the growth, trends, and patterns of scientific research over time. It provides insights into the major contributors, influential journals, top keywords, and research topics that shape the current understanding of a given subject [14,15]. In the case of dry socket, such an analysis can help identify key areas of focus in the literature and highlight the bibliometric parameters. Given this surge in research activity, it has become increasingly important to assess the key attributes of the literature surrounding dry sockets. This bibliometric study aims to fill this knowledge gap by analyzing the publication trends, citation patterns, and other critical factors that define the evolving body of research on dry sockets.

The current bibliometric analysis examines the global research landscape on dry sockets by retrieving relevant publications from the WoS database. The primary objectives of the current study were to analyze the ratio of clinical versus nonclinical studies to examine the level of evidence (LoE), to review the publication trends and citation analysis over time, to highlight the leading journals, and to scrutinize the geographical distribution of research by identifying the top 10 countries and top 10 institutions. The secondary objectives included assessing the top 10 productive authors, their contributions and impacts, and exploring evolving topics by examining the top 20 keywords.

## Review

Methodology

Search Strategy

**Figure 1:**
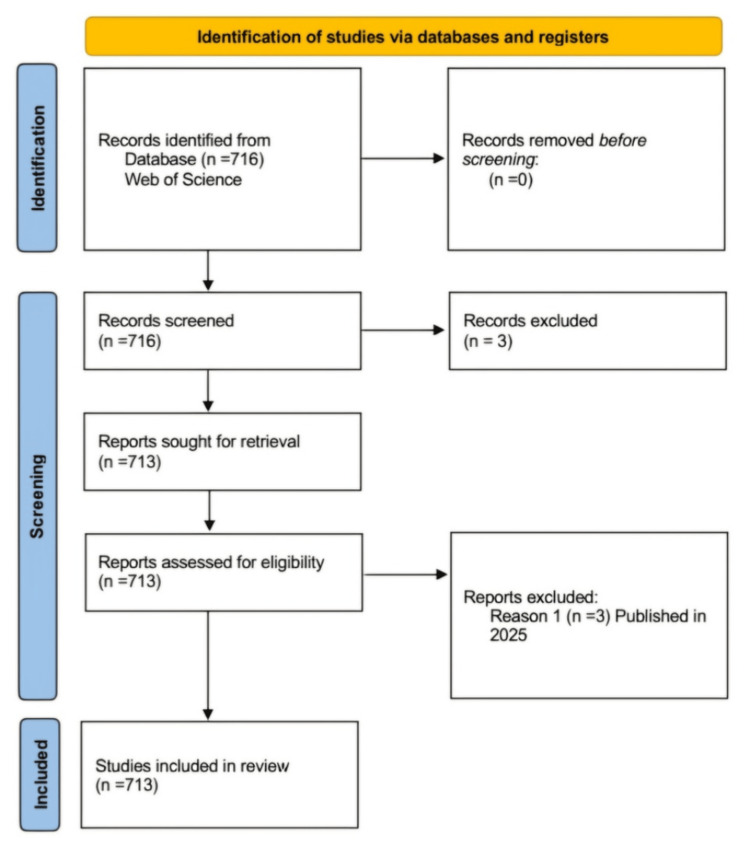
PRISMA flow diagram showing the screening process of articles PRISMA: Preferred Reporting Items for Systematic Reviews and Meta-Analyses Image credit: This is an original image created by the author Manju Philip

The study utilized a quantitative bibliometric approach using the Preferred Reporting Items for Systematic Reviews and Meta-Analyses flow diagram to analyze the dataset retrieved from the Clarivate Analytics WoS database (Philadelphia, PA) on December 31, 2024 (Figure 1). The search strategy included the terms Topics = (“dry socket” OR “alveolar osteitis”). Seven hundred and thirteen studies were included for quantitative synthesis. The initial search yielded 716 documents published between 1905 and 2024, with the year 2025 excluded using a year filter. All publication types were included in the bibliometric analysis. A few records published in 2025 were excluded. Microsoft Excel (version 16, Microsoft Corporation, Redmond, Washington) and VOSviewer (version 1.6.10, Centre for Science and Technology Studies, Leiden University, The Netherlands) were used for data visualization and analysis. The study extracted and analyzed the following data: the distribution of publications by clinical/nonclinical study type, assessment of the LoE, publication and citation trends by time intervals, the leading publishing journals, as well as the top countries, institutions, authors, and 20 most frequently used keywords. The modified Oxford Level of Evidence scale was used to assess the publications based on their LoE. The impact factor of each journal, according to the Journal Citation Reports for the year 2023, indicates the average number of citations to articles published in that journal during the previous two years. Citation impact represents the average number of citations per publication. It is calculated by dividing the total citations by the number of publications, indicating the journal’s overall citation influence.

Ethical Consideration

The present study used publicly available datasets and did not involve human or animal data, so institutional review board approval was not required.

Results

Distribution of Publication by Clinical/Nonclinical Nature of the Study Based on LoE

The study distributed the dry socket publications into the clinical and nonclinical nature of the study and the LoE. Table 1 states that most publications (n = 636; 89%) are clinically related studies, while only 11% (n = 77) of publications belong to nonclinical studies. There is no big difference between the citation impact of clinical and nonclinical studies. The analysis of LoE illustrates that slightly more than two-thirds of the publications (n = 482; 67.60%) are related to LoE-1 and LoE-2. A promising number of publications were found in LoE-4, but few publications were found in LoE-3 and LoE-5. The citation analysis of LoE shows that publications related to LoE-1 gained the highest citation impact, with 22.04 citations per publication, followed by LoE-2, with 20.50 citations per publication. LoE-3 publications gained the lowest citation impact.

**Table 1:** Distribution of publication based on the nature of studies and LoE LoE: level of evidence

Parameters	Variables	Total publications	Total citations	Citation impact
Nature of studies	Clinical studies	636	12,027	18.91
	Nonclinical	77	1,414	18.36
LoE	LoE-1	207	4,562	22.04
	LoE-2	275	5,638	20.50
	LoE-3	46	327	7.11
	LoE-4	117	1,667	14.25
	LoE-5	68	1,247	18.34

Periodic Growth of Publications, Citations, and Citation Impact

**Figure 2:**
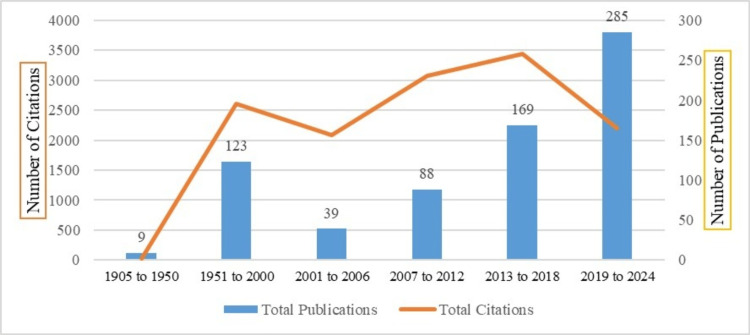
Distribution of publications, citations, and citation impact by time intervals Image credit: This is an original image created by the author Ikram UI Haq

The data in Figure 2 illustrate the periodic growth of publications and citations in the literature on dry sockets, organized by time intervals. We divided the time span into different intervals. The first interval consisted of 1905-1950; during this period, only nine publications were found, and in the next 50 years, from 1951 to 2000, 123 publications were identified. The next 24 years were divided into four intervals containing six years each, and 81.48% of publications were published in these years. There is an obvious escalation in the number of publications over time, especially from the 2001-2006 period onward, with a significant surge in the latest period from 2019 to 2024, where 285 publications were recorded.

The citation metric shows that 713 publications were cited, with an average of 18.85 citations per publication. Citation counts have increased significantly over the years, with peaks in earlier periods (especially 2001-2006 and 2007-2012). However, despite having more publications, the total citations in 2019-2024 (n = 2,197) are slightly lower than in some previous periods.

Citation impact has been calculated as total citations divided by total publications. The highest ratio of citation impact was observed during the 2001-2006 period, with 53.62 citations per publication. This suggests that publications during this time may have been highly influential or extensively cited.

Frequently Used Journals

The literature of 713 publications on dry socket has been published in 219 sources; 59% (n = 129) of the sources published one publication each, and more than one-third (n = 272; 38.14%) of the publications were published in the top 10 journals, as shown in Table 2. The highest number of publications (n = 82) were published in the Journal of Oral and Maxillofacial Surgery, followed by the International Journal of Oral and Maxillofacial Surgery (n = 31). The Journal of Dental Research leads with an impact factor of 5.7. However, it has fewer publications (n = 27), and the journal Oral Surgery, Oral Medicine, Oral Pathology, Oral Radiology, and Endodontology has achieved an exceptionally high citation impact of 52.85 among the top 10 journals.

**Table 2:** Top 10 most frequently used journals JCR: Journal Citation Report

Serial no.	Journal name	Impact factor (JCR 2023)	Total publications	Total citations	Citation impact
1.	Journal of Oral and Maxillofacial Surgery	2.3	82	2,853	34.79
2.	International Journal of Oral and Maxillofacial Surgery	2.2	31	1,045	33.71
3.	Journal of Dental Research	5.7	27	97	3.59
4.	Medicina Oral, Patologia Oral y Cirugia Bucal	1.8	23	398	17.3
5.	British Dental Journal	2.0	22	365	16.59
6.	British Journal of Oral and Maxillofacial Surgery	1.7	22	842	38.27
7.	Oral Surgery, Oral Medicine, Oral Pathology, Oral Radiology, and Endodontology	1.457	20	1,057	52.85
8.	Clinical Oral Investigations	3.1	17	517	30.41
9.	Journal of Maxillofacial and Oral Surgery	0.8	15	89	5.93
10.	Journal of the American Dental Association	3.1	13	242	18.62

Top Countries

The authors from 83 countries had contributed to 713 publications on dry sockets. The authors from 24 countries contributed in one article each. Table 3 presents the details of the top 10 productive countries. The United States leads in both total publications (n = 103) and has the highest citation impact, with an average of 32.06 citations per publication. India comes second in terms of total publications (n = 79) but has a significantly lower citation impact. England produced 61 publications and received 1,813 citations, resulting in a citation impact of 29.72, while Italy contributed 38 publications, yielding an average of 28.97 citations per publication. China has a smaller number of publications (n = 43) but a relatively high citation impact (22.7 cites/pub). Pakistan ranks last with just 22 publications, leading to a very low citation impact (2.27 cites/pub), indicating that the country's publications have relatively low academic influence regarding citations.

**Table 3:** Top 10 productive countries

Rank	Country	Total publications	Total citations	Citation impact
1.	United States	103	3,302	32.06
2.	India	79	656	8.30
3.	England	61	1,813	29.72
4.	China	43	976	22.70
5.	Italy	38	1,101	28.97
6.	Spain	36	793	22.03
7.	Brazil	35	504	14.40
8.	Turkey	28	529	18.89
9.	Iran	28	429	15.32
10.	Pakistan	22	50	2.27

Top Institutions

Eight hundred and sixty-six institutions have been recognized by the VOSviewer software, while the authors affiliated to three-fourths (n = 659; 76%) of the institutions performed research in a single publication. Table 4 presents the details of 10 top-ranked institutions contributing to eight or more than eight publications. The Saveetha Institute of Medical and Technical Sciences emerged as the most productive, with a significant number of publications (n = 22), but these publications gained a modest citation impact (8.50 cites/pub). The University of Manchester leads in terms of citation impact (52.38 cites/pub), with 13 publications, and the University of London follows closely with 43.85 citation impact. The University of Milan has a strong citation impact of 47.00 despite publishing eight papers. Universidade de São Paulo and the University of Hong Kong ranked 10th with identical publication and citation counts, as the eight publications resulted from their collaborative efforts and shared co-authorship.

**Table 4:** Top 10 productive institutions

Rank	Institutions	Total publications	Total citations	Citation impact
1.	Saveetha Institute of Medical and Technical Sciences	22	187	8.50
2.	The University of Manchester	13	681	52.38
3.	University of London	13	570	43.85
4.	Islamic Azad University	10	176	17.60
5.	University of Barcelona	9	250	27.78
6.	Newcastle University	9	232	25.78
7.	University of Milan	8	376	47.00
8.	United States Department of Defense	8	253	31.63
9.	Mashhad University of Medical Sciences	8	210	26.25
10.	Universidade de Sao Paulo	8	177	22.13
11.	University of Hong Kong	8	177	22.13

Top Productive Authors

A total of 2,588 authors were identified in 713 publications, and 89% (n = 2,312) of the authors contributed to a single publication, while 276 authors produced more than one publication. Table 5 presents the details of the top 10 productive authors. Thomas B. Dodson from the University of Washington, Seattle, United States, stands out with the highest citation impact (80), having published six papers. Majid Eshghpour from Mashhad University of Medical Sciences, Iran, follows with a strong citation impact of 29, having published seven papers that received 203 citations. Helen V. Worthington from The University of Manchester has five papers with 160 citations, leading to a citation impact of 32, while H. Brin from Aarhus University has four papers with a high citation impact of 52.25, indicating that his work, though fewer in number, has been highly influential.

**Table 5:** Top 10 productive authors

Rank	Author name	Affiliation	Total publications	Total citations	Citation impact
1.	Majid Eshghpour	Mashhad University of Medical Sciences, Iran	7	203	29.00
2.	Vahid Rakhshan	Islamic Azad University, Iran	7	163	23.29
3.	Thomas B. Dodson	University of Washington, Seattle, United States	6	480	80.00
4.	Massimo del Fabbro	University of Milan, Italy	5	222	44.40
5.	Helen V. Worthington	The University of Manchester, England	5	160	32.00
6.	Paulo S. P.de Carvalho	University of Sao Paulo, Brazil	5	110	22.00
7.	E. Ann Field	University of Liverpool, England	5	73	14.6
8.	Wasiu Lanre Adeyemo	University of Lagos, Nigeria	5	26	5.20
9.	Murugesan Krishnan	Saveetha Institute of Medical and Technical Sciences, India	5	5	1.00
10.	H. Brin	Aarhus University, Denmark	4	209	52.25

Co-Occurrence Network of Keywords

The top 20 keywords and their co-occurrence network were generated using the VOSviewer software. The top 20 keywords related to dry sockets were presented along with two key metrics: occurrences and total link strength. Occurrences indicate how many times a particular keyword appears in the analyzed dataset, reflecting the frequency of that term in relation to dry socket research. Keywords with higher occurrences are likely more central to the topic. The metric of total link strength represents the strength of connections or associations that a particular keyword has with other keywords. A higher link strength suggests the keyword is more interconnected with other terms, indicating its broader relevance or significance in understanding dry sockets. This metric can indicate the depth of a keyword's association with other related topics. “Dry Socket” and “Alveolar Osteitis” appear at the top, as expected, because they are the central concepts. Keywords like “Pain”, “Third Molar”, “Tooth Extraction”, and “Oral Surgery” are also highly ranked, emphasizing the clinical and symptomatic focus of dry socket. Keywords with lower occurrences, like “Coronectomy”, “Hyaluronic Acid”, and “Antibiotics”, show more specific applications or treatments. However, their link strength indicates that they are still relevant in managing or preventing dry sockets.

**Figure 3:**
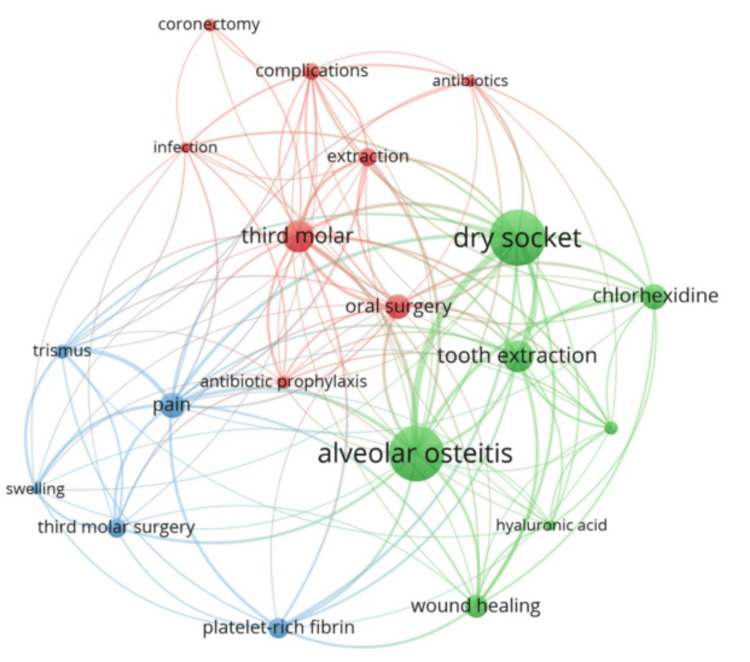
Co-occurrence network of top 20 keywords Image credit: This is an original image created by the author Ikram UI Haq

The co-occurrence network of the top 20 authors used keywords revealed in three distinct clusters generated by the VOSviewer software (Figure 3). The first cluster included eight keywords: antibiotic prophylaxis, antibiotics, complications, coronectomy, extraction, infection, oral surgery, and third molar. The second cluster contained seven keywords: alveolar osteitis, chlorhexidine, dry socket, hyaluronic acid, postoperative complications, tooth extraction, and wound healing. The third cluster comprised five keywords: pain, platelet-rich fibrin, swelling, third molar surgery, and trismus.

Discussion

The current bibliometric study identified 713 publications on dry sockets from 1905 to 2024. The WoS database utilized in this study is an ideal choice for bibliometric analysis because of its comprehensive coverage and robust features. It includes various journals across various fields, including medical and dental sciences. One of its standout features is its extensive citation data, which allows researchers to track how often a particular work is cited, providing insights into its impact and significance within the academic community. Additionally, WoS facilitates interdisciplinary research by indexing publications from multiple disciplines [16,17]. Performing bibliometric analysis with the WoS database is essential for gaining a comprehensive, reliable, and advanced understanding of research trends and their impacts [18].

Our study categorized dry socket publications into clinical and nonclinical studies and LoE. Most publications (89%) are clinical, with a slight difference in citation impact with nonclinical studies. Over two-thirds of publications (n = 482) are related to LoE-1 and LoE-2, with LoE-1 publications gaining the highest citation impact. Chaudhry et al. examined the LoE of 1,300 papers published in the Journal of Maxillofacial and Oral Surgery from 2009 to 2020. Only 7% of the papers published related to LoE-1 and LoE-2, while most of the papers (36%) were related to LoE-5 [19]. Another study examined the LoE on 522 papers published in The Saudi Dental Journal from 2012 to 2021 and found that nonclinical studies accounted for a higher percentage (55.17%) than clinical studies (44.83%). However, clinical studies had a greater proportion of LoE-1 and LoE-2 papers [20].

The findings of our study of periodic growth of publications and citations stated that only nine publications were found in the early 46 years (1905-1950), followed by 123 publications in the next 50 years from 1951 to 2000. Most of the literature (81.48%) was published from 2001 onward. The highest citation impact was found in the 2001-2006 period, with 53.62 citations per publication, suggesting influential publications. The selected publications (n = 713) were cited with an average of 18.85 citations per publication. Although the number of publications has steadily increased over time, the citation impact shows a decreasing trend, suggesting that more recent publications may not be cited as frequently as those from earlier decades. Bibliometric studies typically indicate that older papers have had more time to accumulate citations [21].

Our study revealed that 713 dry socket publications were published in 219 sources, with 59% of the sources publishing one publication each. More than one-third of the publications (38.14%) were published in the top 10 journals. The Journal of Oral and Maxillofacial Surgery had the highest number of publications, followed by the International Journal of Oral and Maxillofacial Surgery. Falci et al. examined the bibliometric traits of the 100 most cited papers in the field of third molar surgery. The study identified the Journal of Oral and Maxillofacial Surgery as the leading journal, with Oral Surgery, Oral Pathology, Oral Radiology, and Endodontology closely trailing behind [8].

The United States emerged as the leading country in extraction and socket preservation research, contributing the highest number of publications and garnering the most citations. This prominence can be attributed to the presence of numerous active research groups and ample funding for research initiatives. India came second in terms of the number of publications, followed by England and Italy, which produced influential publications. Other studies also endorsed that the United States contributed the highest number of papers in oral and maxillofacial surgery [7,8]. The United States has a well-established network of academic institutions, research centers, and specialized dental schools that foster a culture of innovation and collaboration. The United States government also provides significant financial support for dental research [22].

Universities significantly impact society and human existence, propelling advancements in technology and research. Understanding the research conducted by universities and the trends that result from their work is essential [23]. The VOSviewer software identifies 886 institutions, with 76% conducting research in a single publication. The University of Manchester leads in citation impact, followed by the University of London. Other institutions like the University of Milan, the United States Department of Defense, and Mashhad University of Medical Sciences have strong citation impacts. However, while producing a large number of publications, the Saveetha Institute of Medical and Technical Sciences has a comparatively low citation impact, indicating that although the amount of research is substantial, its academic influence may not be as great as that of some of the other institutions on the list.

The study identified 89% of the authors contributing to single publications, and Majid Eshghpour from Mashhad University of Medical Sciences was found to be the most productive, while Thomas B. Dodson from the University of Washington was found to be the most impactful author. This analysis also illustrates the varied academic impact of different researchers, with a notable difference in citation impact despite similar numbers of publications. Some authors, like Thomas B. Dodson and H. Brin, have high citation impacts, suggesting that their research has been widely recognized. In contrast, others like Murugesan Krishnan and Wasiu Lanre Adeyemo have lower citation impacts, which may indicate that their publications are either newer or have not yet gained as much academic traction. In line with these findings, Gutiérrez-Vela et al. evaluated the regenerative periodontal surgery published in 30 years (1980-2010), and the study reported that 4,823 authors contributed to 1,794 documents, and 79.6% of the authors had contributed to just a single document. These findings demonstrate that a small group of researchers contributed the most numbers of research [24].

By analyzing the frequency and distribution of keywords in published literature, researchers can identify emerging trends, key topics, and shifts in the direction of scientific inquiry over time. This process helps assess the current state of a field and anticipate its future developments [25]. The evaluation of the top 20 keywords in our study is grouped into three clusters based on their co-occurrence patterns, highlighting different aspects of the condition: prevention and surgical procedures, postextraction recovery, and symptom management. The keywords “Dry Socket” and “Alveolar Osteitis” dominate the analysis, reflecting their central importance, while other terms like “Coronectomy” and “Hyaluronic Acid” represent more specialized treatments and interventions. The clustering provides a visual representation of the main research themes and can guide further exploration of the key factors involved in dry socket's pathophysiology, treatment, and management [26]. Yeung et al. examined the 79 papers on coronectomy published from 1992 to 2016. The thematic assessment stated that the “nerve” was a key focus in coronectomy research, with terms like “canal”, “nerve”, and “proximity” receiving high citation rates, while postoperative complications, including "dry socket", "infection", and "safety", also attracted significant attention [27]. Yu and Chang evaluated the 562 papers on platelet-rich fibrin in dentistry published from 2006 to 2022. The study reported that the “extraction socket” was the most common site for platelet-rich fibrin application, followed by its use in extraction sockets during oral surgery treatments [28]. Almas et al. examined the literature on tooth extraction socket preservation and highlighted that grafts, extraction sockets, and alveolar ridge augmentation were prevalent keywords in research published during the 20th century [7]. Falci et al. investigated the bibliometric features of third molar surgery. “Surgery”, “removal”, and “complications”, were the top three most occurred keywords [8].

There are a few shortcomings in our study. First, we relied on the dataset retrieved from one source, the WoS database. Although it provides comprehensive coverage of scientific literature, regional, lesser known publications and research published in nonindexed journals may not be included in our dataset. Future studies may combine the data indexed in Scopus, PubMed, Google Scholar, and even gray literature for more in-depth analysis. Second, our study's reliance on citation counts for assessing the impact of publications may introduce a bias toward older or more frequently cited publications, even though these may not necessarily reflect the current trends or innovations in the field. These limitations highlight that while bibliometric studies provide valuable quantitative insights into research trends, they should be interpreted with caution, particularly when assessing the quality, impact, and overall state of research in a given field. Last, our findings are specific to "dry socket" or "alveolar osteitis" and may not be applicable to the broader field of oral and maxillofacial surgery. Future studies exploring other areas within this discipline would offer a more comprehensive understanding of publication trends across the field.

## Conclusions

This bibliometric analysis provides a comprehensive overview of the research landscape on dry sockets, revealing significant trends and key findings. Most of the studies (89%) focused on clinical aspects, with a notable preponderance of publications at LoE-1 and LoE-2, indicating a strong clinical orientation in dry socket research. The study highlighted the rapid growth in the volume of publications, with a sharp increase in research output since 2001, especially from 2019 to 2024. In terms of publishing outlets, the Journal of Oral and Maxillofacial Surgery led in the number of dry socket-related publications, followed by other well-regarded journals such as International Journal of Oral and Maxillofacial Surgery. Geographically, the United States emerged as the leading contributor to dry socket research, both in terms of the number of publications and citation impact. Other countries such as India, England, and China also made significant contributions, although their citation impact varied. The analysis of the top 20 keywords revealed that “dry socket” and “alveolar osteitis” were central themes, with terms related to clinical symptoms and treatments such as “pain”, “tooth extraction”, and “oral surgery” also appearing prominently. Overall, this study provides valuable insights into the key contributors, journals, and research topics in dry socket, and suggests that future research should focus on innovative treatments, prevention strategies, and underexplored areas.
